# Traditional pediatric massage enhanced the skeletal muscle mass in OVA-exposed adolescent rats via regulating SCFAs-FFAR2-IGF-1/AKT pathway

**DOI:** 10.3389/fmicb.2024.1492783

**Published:** 2025-01-03

**Authors:** Lin Lin, Siyuan Li, Que Liu, Xingxing Zhang, Ying Xiong, Shaoyun Zhao, Liyue Cao, Jiaxuan Gong, Yaping Liu, Rong Wu

**Affiliations:** ^1^Department of Traditional Chinese Medicine, Shijiazhuang Medical College, Shijiazhuang, Hebei, China; ^2^College of Acupuncture Moxibustion and Tuina, Nanjing University of Chinese Medicine, Nanjing, Jiangsu, China; ^3^Department of Acupuncture Moxibustion, Nantong First People's Hospital, Nantong, Jiangsu, China; ^4^Department of Medicine, Qinghai University, Xining, Qinghai, China

**Keywords:** massage, adolescent, asthma, skeletal muscle, gut microbiota, short-chain fatty acids

## Abstract

**Objective:**

This study aimed to investigate the potential relation between the retarded growth of skeletal muscle (SM) and dysbiosis of gut microbiota (GM) in children with asthma, and to explore the potential action mechanisms of traditional pediatric massage (TPM) from the perspective of regulating GM and short-chain fatty acids (SCFAs) production by using an adolescent rat model of asthma.

**Methods:**

Male Sprague-Dawley rats aged 3weeks were divided randomly into the 5 groups (n=6~7) of control, ovalbumin (OVA), OVA + TPM, OVA + methylprednisolone sodium succinate (MP) and OVA + SCFAs. Pulmonary function (PF) was detected by whole body plethysmograph, including enhanced pause and minute ventilation. Airway allergic inflammation (AAI) status was assessed by concentrations of OVA-specific immunoglobulin E in plasma, interleukin (IL)-4 and IL-1β in bronchoalveolar lavage fluid via ELISA assay. SM mass was assessed by using cross-sectional areas of diaphragm muscle and gastrocnemius via hematoxylin and eosin staining. GM and SCFAs production were detected by 16S rDNA sequencing and GC-MS, respectively. The protein and gene expressions of free fatty acid receptor 2 in SM were detected by using immunohistochemical staining and qRT-PCR, respectively. qRT-PCR was used to detect other relative gene expressions that were closely related with SM mass. The activity of insulin-like growth factor-1 (IGF-1)/protein kinase B (PKB/AKT) pathway in SM was detected by western blotting test.

**Results:**

OVA exposure caused obvious AAI and poor PF in adolescent rats. OVA-exposed adolescent rats had a retarded growth of SM mass and inhibited activity of IGF-1/AKT pathway, which was related with GM dysbiosis, reduced SCFAs production and FFAR2 expressions in SM. TPM efficiently enhanced the SM mass, along with alleviating AAI and improving PF. TPM activated IGF-1/AKT pathway in SM, which was closely related with correcting GM dysbiosis, enhanced SCFAs production and FFAR2 expressions.

**Conclusion:**

The retarded growth of SM mass and inhibition of IGF-1/AKT pathway existed in OVA-exposed adolescent rats, which was related with GM dysbiosis, reduced SCFAs production and FFAR2 expressions in SM. TPM efficiently enhanced the SM mass, at least, partially via regulating GM, enhancing SCFAs production and activating FFAR2-IGF-1/AKT pathway.

## 1 Introduction

Asthma is characterized by chronic airway inflammation, airway hyperresponsiveness (AHR), variable and later even persistent expiratory airflow limitation, which is the most common chronic disease in school-aged children and often begins in early childhood (Lizano-Barrantes et al., 2024; GINA, 2024). According to the latest global report, only 44.1% of children and 55.4% of adolescents with asthma were well controlled (García-Marcos et al., 2023).

Childhood and adolescence are critical periods for skeletal muscle (SM) growth and development (Orsso et al., 2019; Faienza et al., 2023). However, children with poorly controlled asthma are often accompanied by a decline in SM mass and strength, which may become worse after repeated systematic or even inhaled application of glucocorticoids (GCs), since GCs is the first-line medication for asthma control (Villa et al., 2011; Latorre-Román et al., 2014; Papurcu et al., 2021; Silva et al., 2021; Zhang et al., 2022; Gokcek et al., 2023; Kankaanranta and Ilmarinen, 2023). It indicates that asthma children are susceptible to a retarded growth of SM mass. As a major motor organ, SM also involves in pulmonary function (PF) and metabolism regulation (Frontera and Ochala, 2015; Argilés et al., 2016). Poor strength and endurance of respiratory muscle further impairs PF and worsen the symptoms of chronic respiratory diseases (McKenzie et al., 2009; Lista-Paz et al., 2023). PF in asthmatic children can be improved by strengthening respiratory muscle, especially diaphragm muscle (DIAm) (Elnaggar et al., 2023; Lista-Paz et al., 2023). The SM mass is positively correlated with SM strength (Lanza et al., 2022, 2023). Mounting evidences prove that poor PF is related with declined SM mass and strength in children, all of which are closely associated with poor prognosis of respiratory diseases, increased risks of metabolic syndrome and cardiovascular diseases, and impaired development of skeleton and neurocognition (Kim and Valdez, 2015; Kim and Park, 2016; Agustí et al., 2017; Vasquez et al., 2017; Melén et al., 2019). Therefore, enhancing SM mass is essential to children asthma control and general health.

During asthma development, several factors may negatively influence SM mass, such as inflammation, reduced physical exercise, systematic application of GCs, dysbiosis of gut microbiota (GM) (Schiaffino et al., 2013; Vasquez et al., 2017; Lahiri et al., 2019). Properly exercise can enhance SM mass, improve PF and thus help control childhood asthma (Wanrooij et al., 2014). However, exercises may also speed up inhaling air and directly trigger airway contraction and aggravate inflammation once the inhaled air is not sufficiently warmed and humidified (Bonini and Silvers, 2018). Therefore, how to properly exercise is always a question for asthmatic children.

It is well known that GM dysbiosis involves in the development of children asthma (Stiemsma and Turvey, 2017; Zimmermann et al., 2019). Many interactions between the host and GM are mediated by short-chain fatty acids (SCFAs), which are generated from GM fermentation of dietary polysaccharides (Lahiri et al., 2019). Acetate, propionate and butyrate account for 90~95% of the total SCFAs in the colon, which can reach various organs to exert physiological functions via blood circulation (Trompette et al., 2014). GM diversity is essential for SCFAs production, and the reduced production of SCFAs contributes to skewed Th2 response and asthma development (Cummings et al., 1987; Bauer and Thiele, 2018; Patrick et al., 2020).

Recently, a series of studies strongly support the association of GM and SM mass (Lahiri et al., 2019; Lv et al., 2021). Studies indicated that SCFAs could activate free fatty acid receptor 2 (FFAR2, also called GPR43) and protein kinase B (PKB, more often called AKT) signaling to improve muscular atrophy in mice of diabetic nephropathy model (Huang et al., 2020; Tang et al., 2022). FFAR2 is a primary receptor of all SCFAs, which is expressed in various tissues and organs (Milligan et al., 2009; Huang et al., 2020; Tang et al., 2022). Previous studies also indicated that GM dysbiosis could reduce insulin-like growth factor-1 (IGF-1) expression as well as decrease the mass of SM and bone (Lahiri et al., 2019; Lv et al., 2021). The activation of IGF-1/AKT pathway is essential to the growth or maintenance of SM mass, which not only triggers protein synthesis but inhibits protein degradation via different downstream pathways (Stitt et al., 2004; Sandri, 2013). Therefore, these evidences above suggest that the retarded growth of SM mass in asthmatic children may be related to GM dysbiosis, reduced SCFAs-FFAR2 levels and inhibited IGF-1/AKT pathway, which can be corrected by regulating GM and enhancing SCFAs production.

Atopy is defined as a personal and/or familial propensity to produce IgE antibodies and sensitization in response to environmental triggers. Underlying atopy has been considered to be critical in linking asthma and other atopic diseases, all of which are strongly influenced by genetic factors. Therefore, it is important to provide a critical window of opportunity early in life for intervention when identify any risk for developing asthma (Zheng et al., 2011; Goksör et al., 2016). Meanwhile, prevention before diseases' occurrence and aggravation is also an important concept in TCM. Preventative Intervention can also be practiced to deter the development and aggravation of children asthma in TCM concept.

Massage has long been applied to prevent and treat children asthma in China and in Western countries (Wu et al., 2017; Kliegman and Geme, 2020). The results of meta-analysis and systematic reviews support massage application on children asthma in the attack and non-attack stages (Wu et al., 2017; Yue et al., 2019). Traditional pediatric massage (TPM) in China always includes several manipulations that produce gentle and moderate stimulations on the back, such as back pinching and pushing manipulations. Traditional Chinese medicine (TCM) believes that Du vessel and bladder meridian on the back can be stimulated to regulate visceral functions and thus boost the body's inherent resistance to diseases (Wu et al., 2024). Our previous clinical trials showed that TPM improved the atopic manifestations and GM dysbiosis in children (Xiong et al., 2017; Liu et al., 2019; Lin et al., 2022).

Our exploratory studies demonstrated that TPM application resisted airway allergic inflammation (AAI) and GM dysbiosis during asthma modeling in adolescent rats (Zhu et al., 2020; Liu et al., 2022). The finding indicated that TPM application might deter the development of children asthma as preventative intervention. To further explore the preventative mechanism of TPM, we design this study to investigate the prevention and treatment of TPM on SM during asthma modeling in adolescent rats, controlled with MP treatment and SCFAs supplement. More importantly, we design this study to explore the potential mechanism on the retarded growth of SM mass in asthmatic children and the action mechanism of TPM from the perspective of regulating SCFAs-FFAR2-IGF-1/AKT pathway by using an adolescent rat model of asthma.

## 2 Materials and methods

### 2.1 Animals

Male specific pathogen-free Sprague-Dawley rats (aged 3weeks, 40~50 g, provided by Shanghai Slack Laboratory Animal Co. LTD) were housed in a standard facility (22 ± 2°C, 30%~40% humidity, a 12 h light/dark cycle) with *ad libitum* access to water and food. After 3-day acclimatization, all rats were divided randomly into the 5 groups (n=6~7) of control (CON), ovalbumin (OVA), OVA + TPM, OVA + methylprednisolone sodium succinate (MP) and OVA + SCFAs (SC). The animal protocol was approved by the Animal Care and Use Committee of Nanjing University of Chinese Medicine (No 202109A043) and carried out in accordance with the National Institutes of Health Guidelines for the Care and Use of Laboratory Animals.

### 2.2 Group treatment

**Figure 1:**
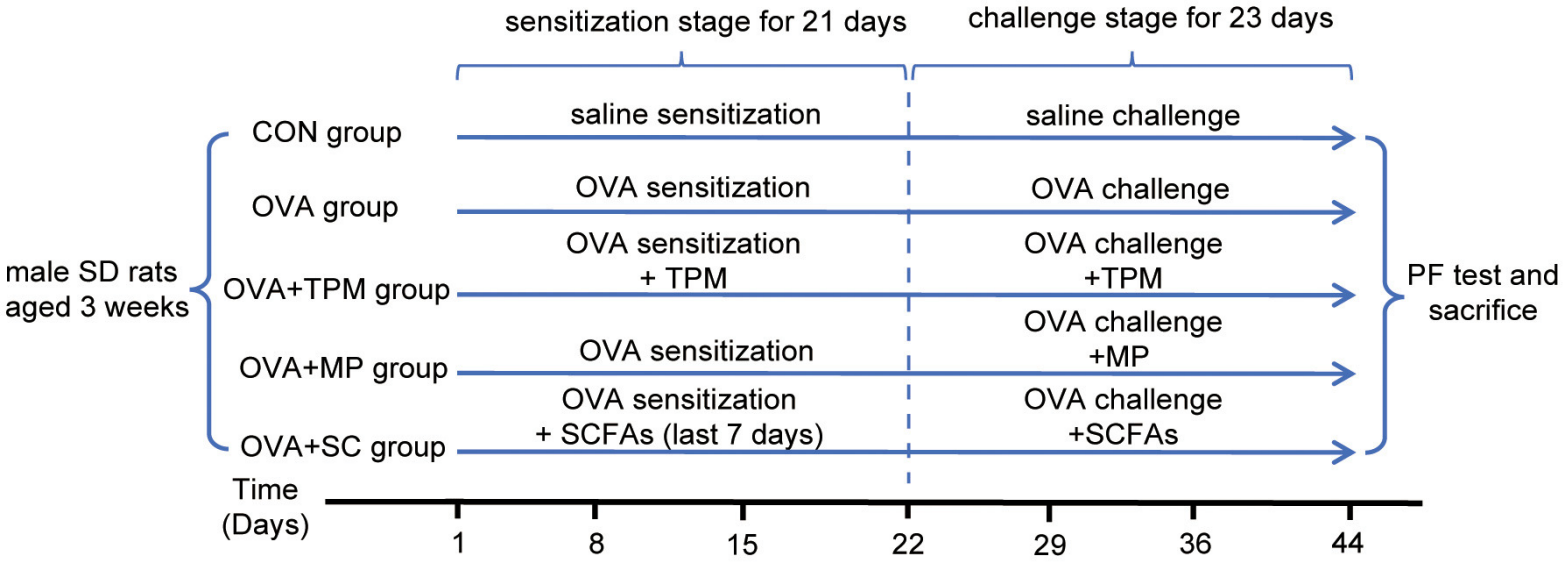
Experiment protocol. OVA, ovalbumin; PF, pulmonary function; MP, methylprednisolone sodium succinate; SCFAs, short chain fatty acids; TPM, Traditional pediatric massage.

All the rats, except the CON group, were given OVA exposure, i.e., OVA sensitization and challenge. The rats were sensitized on day 1, 8 and 15 via intraperitoneal injections (ip) of 2 mg OVA (Grade V, Sigma, No A5503) mixed into 1 mL 4% Al(OH)_3_ gel (BS010, Shanxi ZHHC Biomedical Technology Co., LTD, China). From day 22 to 44, the rats were challenged with 2% OVA (Grade II, Sigma, No A5253) aerosol every other day and with 3% OVA aerosol at the last 2 days for 30 min in a plastic chamber connected to an atomizer (402AI type, Jiangsu Yuwell Medical Instruments Co., Ltd., China). The CON group were exposed with saline for control. The experiment protocol is shown in Figure 1.

Besides OVA exposure, the OVA+TPM group also received TPM once every day from day 1 to 44 at a fixed time (about 30 min before OVA exposure). TPM was conducted by a trained manipulator based on our previous protocol (Zhu et al., 2020; Liu et al., 2022): briefly, step 1, gently held a rat in the left palm and applied pushing manipulation with right fingers on its back for 5 times (speed: ~5 cm/s, pressure: ~5 N detected by Finger TPS system, Pressure Profile System, Inc, USA); step 2, pinched the back skin with the first 3 fingers at the bottom and continuously pushed up to the shoulder level along the middle line and for 15 repeats (~10 s/repeat, pressure: ~12 N) from day 1 to 21 and for 20 repeats from day 22 to 44 to avoid stimulation adaptation; step 3, repeated step 1. The whole procedure avoided any obvious discomfort or screaming in rats. The rest groups were put individually in clean cages and grabbed gently several times for control.

Besides OVA exposure, the OVA+MP group were also given a commonly-used GC medication, MP (ip, 10 mg/kg, 2 mg/mL prepared with saline) (Liu et al., 2022), 30 min before OVA challenge from day 22 to 44. The OVA+SC group were also treated with a cocktail of SCFAs (67.5 mM sodium acetate, 25.9 mM sodium propionate and 40 mM sodium butyrate) in their drinking water (Lahiri et al., 2019), which began from day 15 to 44. No obvious decrease in water intake was noted in the OVA+SC group.

### 2.3 PF measurement

Whole body plethysmograph is widely used for PF assessment in an awake and conscious condition without inducing significant injury or distress in murine (Vaickus et al., 2010). Twenty-four hours after the last OVA exposure, rats were placed in unrestrained chambers and exposed to a 2-min aerosolization of PBS, 3.125, 6.25, 12.5, 25, 50 mg/mL acetylcholine (Ach) respectively, followed by a 5-minute recording (Buxco Research Systems, Wilmington, NC). To evaluate PF status, enhanced pause (Penh) and minute ventilation (MV) were calculated automatically to assess AHR and the volume of air exchange per minute, respectively.

### 2.4 Concentrations of OVA-specific immunoglobulin E (sIgE), interleukin-4 (IL-4) and IL-1β measured by enzyme-linked immunosorbent assay (ELISA)

All rats were deeply anesthetized with 20% Urethane (ip, 5 mL/kg). The plasma and bronchoalveolar lavage fluid (BALF) were collected following previous protocols (Zhu et al., 2020; Liu et al., 2022). To investigate AAI condition, the concentrations of plasma OVA-sIgE and BALF IL-4 and IL-1β were measured by using ELISA kits following the manufacturer's protocol (YJ003273, YJ102825 and YJ037361, Yanju Biotechnology, Shanghai, China).

### 2.5 Cross-sectional areas (CSAs) assessment of SM by hematoxylin and eosin (HE) staining

CSAs of muscle fibers can represent SM mass (Sun et al., 2021; Ribeiro et al., 2023). Both of respiratory and locomotor muscles are SM. Therefore, the DIAm and gastrocnemius (GA) were collected to test as typical respiratory and locomotor muscles in this study. Halves of them were stored in 4% paraformaldehyde at least 48 h. The specimens of the DIAm and GA were dehydrated, embedded in paraffin and then cut into 6-μm-thick sections at the fixed middle part. Sections were stained with HE for observation and measurement. Every sample was observed under a fluorescence optical microscope (DP70, Olympus, Tokyo, Japan) and photographed for three different microscopic fields (×200). For each sample, 100 fibers were randomly chosen by a blind assessor to calculate CSAs by using Image-Pro Plus 6.0 and the average values were used for analysis.

### 2.6 GM analysis by 16s rDNA sequencing

Two fresh fecal pellets from the same segment of the colon were collected and stored at −80°C for 16 S rDNA and gas chromatography-mass spectrography (GC-MS) detection. Fecal pellets were homogenized by using a beadbeating method and total DNA was extracted with E.Z.N.A. Soil DNA Kit (Omega Bio-tek, Inc., USA) following the manual. Purity and quality of the genomic DNA were checked by NanoDrop 2000 spectrophotometer (Thermo Scientific Inc., USA). The V3-4 hypervariable region of bacterial 16S rRNA gene were amplified with the universal primer 338F (5′-ACTCCTACGGGAGGCAGCAG-3′) and 806R (5′-GGACTACNNGGGTATCTAAT-3′). PCR products were mixed in equidensity ratios, which then were purified by using a Agencourt AMPure XP Kit (Beckman Coulter, Inc., USA). Sequencing libraries were generated by using NEB Next Ultra II DNA Library Prep Kit (New England Biolabs, Inc., USA) following the manufacturer's protocol. The library quality was assessed by Nanodrop 2000 (ThermoFisher Scientific, Inc., USA), Agilent 2100 Bioanalyzer (Agilent Technologies, Inc., USA) and ABI StepOnePlus Real Time PCR System (Applied Biosystems, Inc., USA). Finally, the library was sequenced on Illumina Miseq/Novaseq (Illumina, Inc., USA) platform, and 300-bp paired-end reads were generated.

### 2.7 SCFAs concentrations detected by GC-MS

The concentrations of SCFAs in the feces were measured by using Agilent 7890B GC-MS with a DB-FFAP column (30 m × 0.25 mm × 0.25 μm, J&W Scientific, USA). All of the standards were purchased from CNW (Beijing) or Aladdin (Shanghai). The stock solutions of standards were prepared in 1 mg/mL with methyl tert-butyl ether (MTBE, CNW Technologies, Germany), stored at −20°C and further diluted with MTBE to working solutions for analysis. MilliQ water (Millipore, Bradford, USA) was used in the detection. Briefly, fecal samples (20 mg) were homogenized in 1 mL of phosphoric acid (0.5% v/v) solution; supernatant (0.1 mL) was added to centrifugal tube (1.5 mL) after the mixture was centrifuged (12,000 r/min) for 10 min at 4°C; MTBE solution (0.5 mL, containing internal standard) was added; the mixture was vortexed for 3 min and ultrasonicated for 5 min, followed by centrifugation (12,000 r/min) for 10 min at 4°C. The supernatant was collected for GC-MS (GC2030-QP2020 NX, Japan) analysis.

### 2.8 FFAR2 protein expressions in SM detected by immunohistochemical (IHC) staining

Briefly, the paraffin sections of DIAm and GA were deparaffinized for antigen retrieval, subjected to antigen repair, serum-blocked, incubated by using primary antibody FFAR2 (1:250, bs-13536R, Bioss) overnight at 4°C, then incubated by secondary peroxidase-labeled sheep anti-rabbit IgG (SA1022, BOSTER) for 30 min at 37°C, washed with PBS for 5 min 3 times and added with DAB color developing solution (KGP1045-100, KeyGEN). Every sample was observed by a blind observer under a fluorescence optical microscope (DP70, Olympus, Tokyo, Japan) and photographed for 3–5 different microscopic fields (×400). For the brown stained area in each sample, the mean optical density (MOD) was valued by using Image-Pro Plus 6.0 and averaged for analysis.

### 2.9 Gene expressions in SM detected by quantitative real-time polymerase chain reaction (qRT-PCR)

Muscle atrophy F-box (MAFbx, also called atrogin-1) and muscle-specific RING finger protein 1 (MuRF1) are major atrophy-related E3 ubiquitin ligases in the ubiquitin-proteasome system (UPS), which trigger proteasome-dependent degradation (Schiaffino et al., 2013; Yin et al., 2021). Myogenic differentiation (MyoD) and myogenin (MyoG) are major myogenic transcription factors for the protein synthesis (Frontera and Ochala, 2015). The gene expressions of the above molecules and FFAR2 were detected by qRT-PCR in this study. Halves of DIAm and GA were collected and frozen immediately in liquid nitrogen and stored at −80°C. The total RNA was extracted by using a Trizol reagent following the supplier's instruction (Invitrogen Life Technologies, Carlsbad, CA, USA). cDNA was obtained by reverse transcription. Gene expressions were normalized to GAPDH and then to control for analysis. The forward and reverse primers are shown in Table 1.

**Table 1:** Primer sequences for the target genes.

**Gene**	**Forward Primer (5^′^-3^′^)**	**Reverse PRIMER (5^′^-3^′^)**
GAPDH	CTCTCTGCTCCTCCCTGTTC	CGATACGGCCAAATCCGTTC
Atrogin-1	CAACAGACTGGACTTCTCGAC	GAAGTTCTTTTGGGCGATGC
MuRF1	CCCCTTACAAAGCATCTTCCA	TGTTTTCCTTGGTCACTCGG
MyoD	TGCTCTGATGGCATGATGGAT	AGATGCGCTCCACTATGCTG
MyoG	CACAAGCCAGACTACCCACC	CCCGTTGAGGGGCATTAACA
FFAR2	GACCAAGACAGACAGGGGTG	ACCTAGACGCTCGGGAAGAT

MuRF1, Muscle-specific RING finger protein 1; MyoD, myogenic differentiation; MyoG, myogenin; FFAR2, free fatty acid receptor 2.

### 2.10 Western blotting test

The tissues of DIAm and GA were homogenized in an assay lysis buffer (P0013B, beyotime, China) containing phosphatase and protease inhibitors. The blots were blocked for 20 min at room temperature and incubated overnight at 4°C with the primary antibodies, including IGF-1 (1:1,000, 28530-1-AP, Proteintech, China), AKT (1:1,000, T55561, Abmart, China), p-AKT (1:1,000, T40067, Abmart, China) and GAPDH (1:10,000, 60004-1-lg, Proteintech, China), respectively. The membrane was further incubated for 1h at room temperature with a horseralized perxidase-conjugated secondary antibodies, including HRP-conjugated affiniPure goat anti-rabbit IgG (1:10,000, SA00001-2; 1:8,000, SA00001-1; Proteintech, China), respectively. The intensity values of the bands were quantified by using ImageJ software (NIH, Bethesda, USA) and then normalized to GAPDH and to control for analysis.

### 2.11 Statistical analyses

For GM analysis, sequences were analyzed by using the QIIME (v1.8.0) software. Use Pear (v0.9.6) software to filter and splice raw data. Qualified sequences were clustered into operational taxonomic units (OTUs) at a similarity threshold of 97% by using Uparse algorithm of Vsearch (v2.7.1) software. Alpha-diversity indices were also calculated by using QIIME (v1.8.0). Shapiro-Wilk test was used to assess the normality of the data. Normally distributed data are represented as mean ± standard deviation (SD) and analyzed by one-way analysis of variance (ANOVA) with further LSD or Dunnett's T3 *post-hoc* test based on homogeneity of variances. Repeated-measures ANOVA was used for PF data. Non-normally distributed data are represented as interquartile range and analyzed by using Kruskal-Wallis test. SPSS 24.0 (IBM SPSS Inc., Armonk, USA) and GraphPad Prism 6.0 (GraphPad Software Inc., San Diego, USA) software were used for statistical analysis and graphing, respectively. *P* < 0.05 was considered statistically significant.

## 3 Results

### 3.1 OVA exposure induced AAI and poor PF, along with GM dysbiosis and decreased SCFAs concentrations in adolescent rats

**Figure 2:**
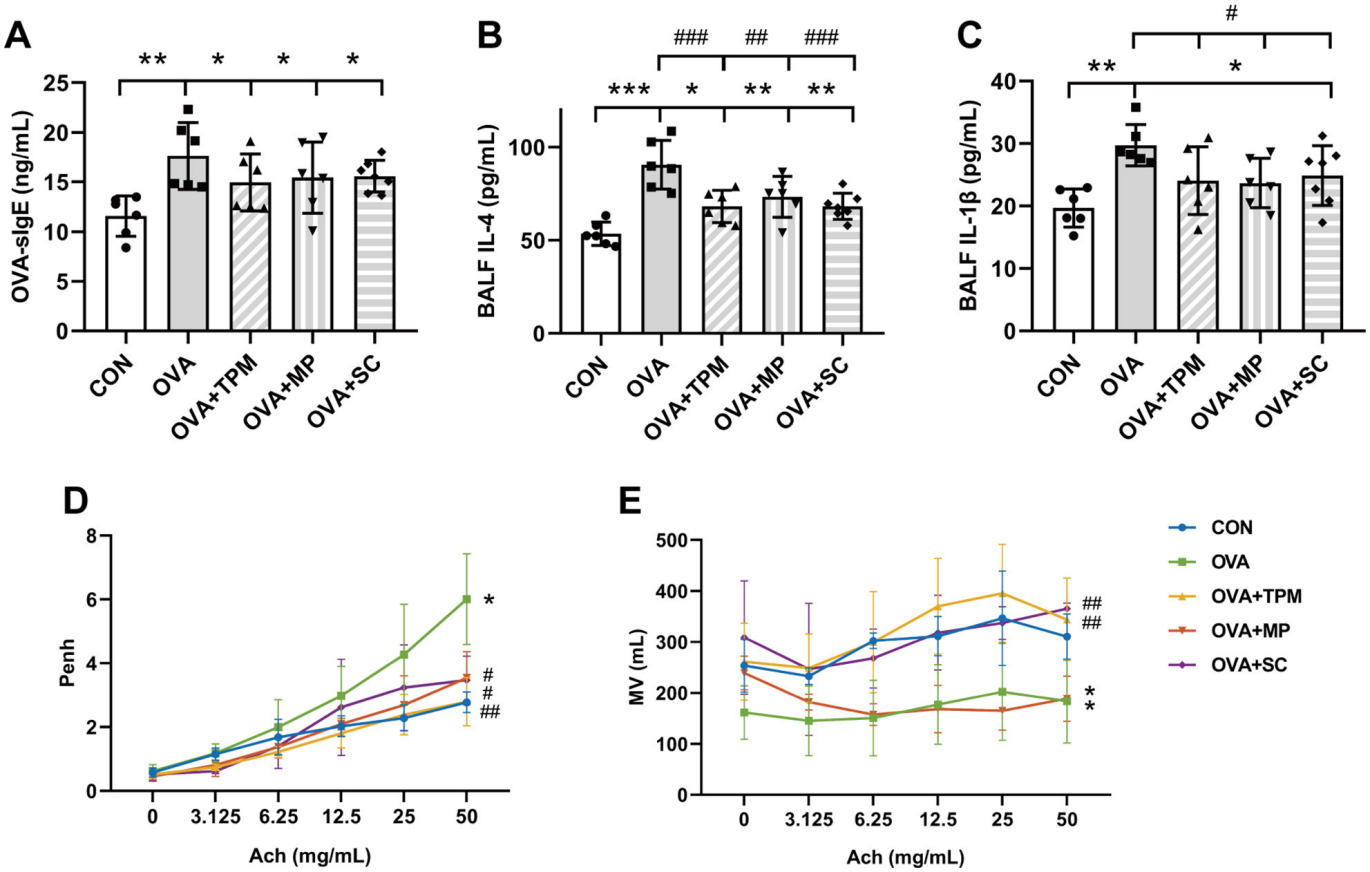
Conditions of airway allergic inflammation and pulmonary function. **(A)** concentration of OVA-sIgE in plasma assessed by ELISA. **(B, C)** concentrations of IL-4 and IL-1β in BALF assessed by ELISA. **(D, E)** PF detected by WBP including Penh value and MV value after Ach challenge. All data are expressed as mean ± SD (*n* = 6 for ELISA; *n* = 3 for PF). *P < 0.05, **P < 0.01, ***P < 0.001, compared with the CON group. ^#^*P* < 0.05, ^##^*P* < 0.01, ^###^*P* < 0.001, compared with the OVA group. CON, control; OVA, ovalbumin; TPM, traditional pediatric massage; MP, methylprednisolone sodium succinate; SC, short chain fatty acids; BALF, bronchoalveolar lavage fluid; sIgE, specific immunoglobulin E; IL, interleukin; PF, pulmonary function; WBP, whole body plethysmograph; Penh, enhanced pause; Ach, acetylcholine; Penh, enhanced pause; MV, minute ventilation; ELISA, enzyme-linked immunosorbent assay; PF, pulmonary function.

Our previous study demonstrated that OVA exposure induced AAI in adolescent rats, along with reduced richness and diversity of GM, decreased butyrate-producing bacterial taxa and abnormally elevated abundance of Lactobacillus (Zhu et al., 2020; Liu et al., 2022). In this study, OVA exposure also successfully induced asthma in adolescent rats. Compared with the CON group, OVA-exposed rats had obvious AAI, manifested as higher concentrations of OVA-sIgE, IL-4 and IL-1β (*P* < 0.01, *P* < 0.001 and *P* < 0.01; Figures 2A–C). Meanwhile, OVA exposure led to poor PF, characterized by significantly higher Penh and lower MV values (both *P* < 0.01; Figures 2D, E).

**Figure 3:**
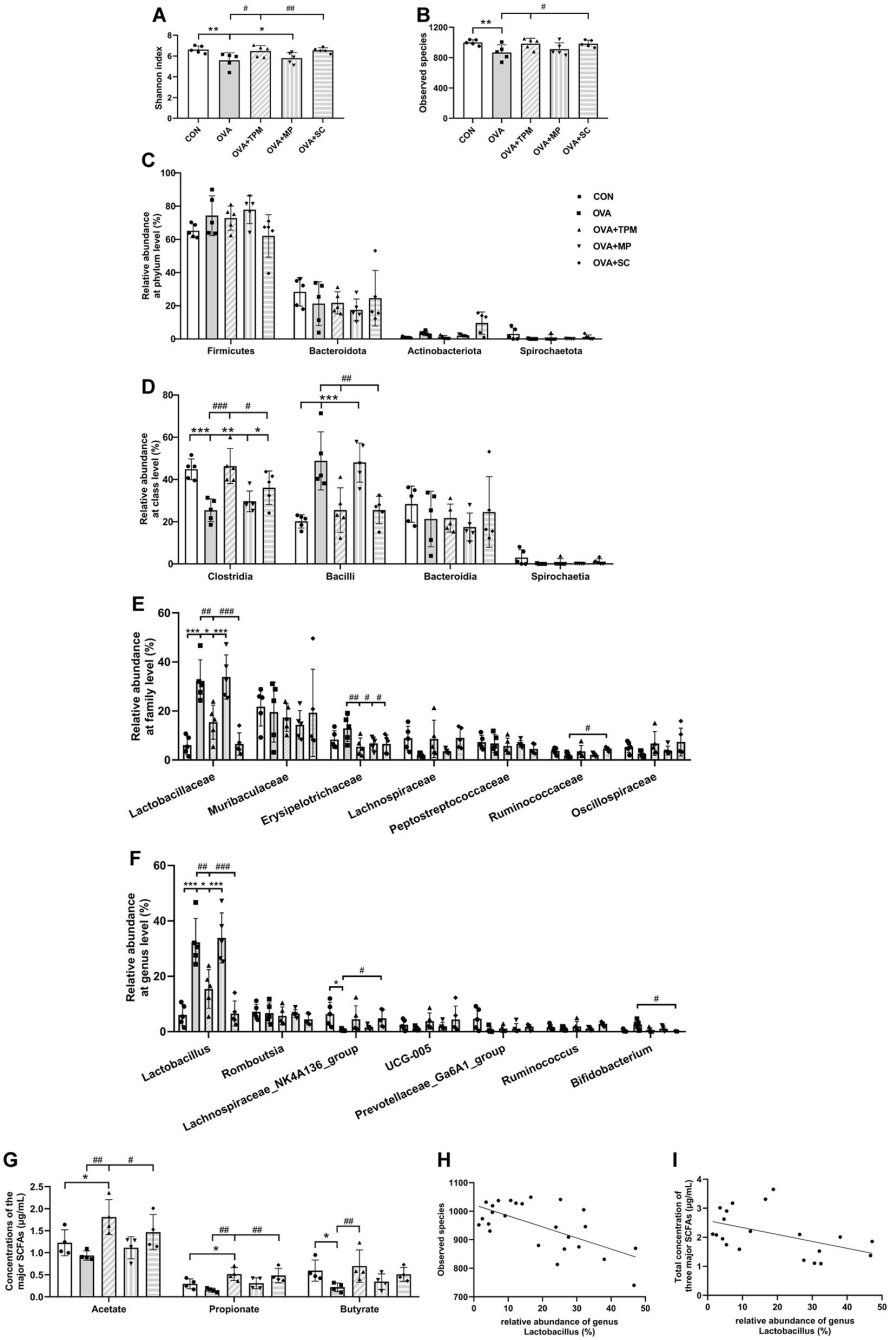
Analysis of GM and SCFAs concentrations. **(A, B)** shannon index and observed species of GM. **(C–F)** relative abundance at the phylum, class, family and genus levels. **(G)** concentrations of acetate, propionate and butyrate. **(H)** correlation analysis of genus Lactobacillus and observed species of GM. **(I)** correlation analysis of genus Lactobacillus and the total concentration of three major SCFAs. All data are expressed as mean ± SD (*n* = 5 for GM; *n* = 4 for SCFAs). ^*^P < 0.05, ^**^P < 0.01, ^***^P < 0.001, compared with the CON group. ^#^*P* < 0.05, ^##^*P* < 0.01, ^###^*P* < 0.001, compared with the OVA group. CON, control; OVA, ovalbumin; TPM, traditional pediatric massage; MP, methylprednisolone sodium succinate; SC, short chain fatty acids; GM, gut microbiota; SCFAs, short chain fatty acids.

We further investigated GM status and SCFAs production in these rats. Compared with the CON group, OVA exposure induced GM dysbiosis in adolescent rats. Firstly, OVA-exposed rats had significantly lower Shannon index and fewer observed species (both *P* < 0.01; Figures 3A, B), which represent the richness and diversity of GM; secondly, OVA exposure didn't induce any significant change in the relative abundances of the major phyla (all *P* > 0.05; Figure 3C), however, OVA exposure significantly decreased class Clostridia abundance and increased class Bacilli abundance (both *P* < 0.001; Figure 3D), both of which belong to phylum Firmicutes; thirdly, OVA exposure induced significantly higher abundance of family Lactobacillaceae and genus Lactobacillus (both *P* < 0.001; Figures 3E, F), which belong to class Bacilli. Then, we investigated the concentrations of acetate, propionate and butyrate, the major SCFAs. The results showed that OVA exposure caused the significantly lower concentrations of acetate, propionate and butyrate (all *P* < 0.05; Figure 3G). These results suggest that asthma development was accompanied by GM dysbiosis and decreased SCFAs production. We further performed the correlation analysis between the abundance of genus Lactobacillus and the observed species of GM as well as the correlation analysis between the abundance of genus Lactobacillus and the total concentration of the three major SCFAs, both of which showed significantly negative correlations (*P* < 0.001 and *P* < 0.05; Figures 3H, I).

### 3.2 TPM prevented GM dysbiosis and enhanced SCFAs concentrations in OVA-exposed adolescent rats, along with improving AAI and PF

Compared with the OVA group, TPM significantly decreased IL-4 and IL-1β concentrations (*P* < 0.001 and *P* < 0.05; Figures 2B, C), improved PF, i.e. reducing the Penh and increasing MV values (both *P* < 0.01; Figures 2D, E) in OVA-exposed adolescent rats, despite non-significantly decreasing OVA-sIgE concentration (*P* > 0.05; Figure 2A). Meanwhile, compared with the OVA group, TPM prevented the decrease in Shannon index and observed species of GM (both *P* < 0.05; Figures 2A, B) and significantly increased class Clostridia abundance (*P* < 0.001; Figure 3D), decreased class Bacilli abundances (*P* < 0.01; Figure 3D) and decreased the abnormally high abundances of family Lactobacillaceae and genus Lactobacillus (both *P* < 0.01; Figures 3E, F). Moreover, TPM significantly increased the production of the major three SCFAs (all *P* < 0.01; Figure 3G), compared with the OVA group.

### 3.3 TPM enhanced the growth of SM mass and abnormal expressions of relative genes induced by OVA exposure in adolescent rats, along with activating FFAR2-IGF-1/AKT pathway

**Figure 4:**
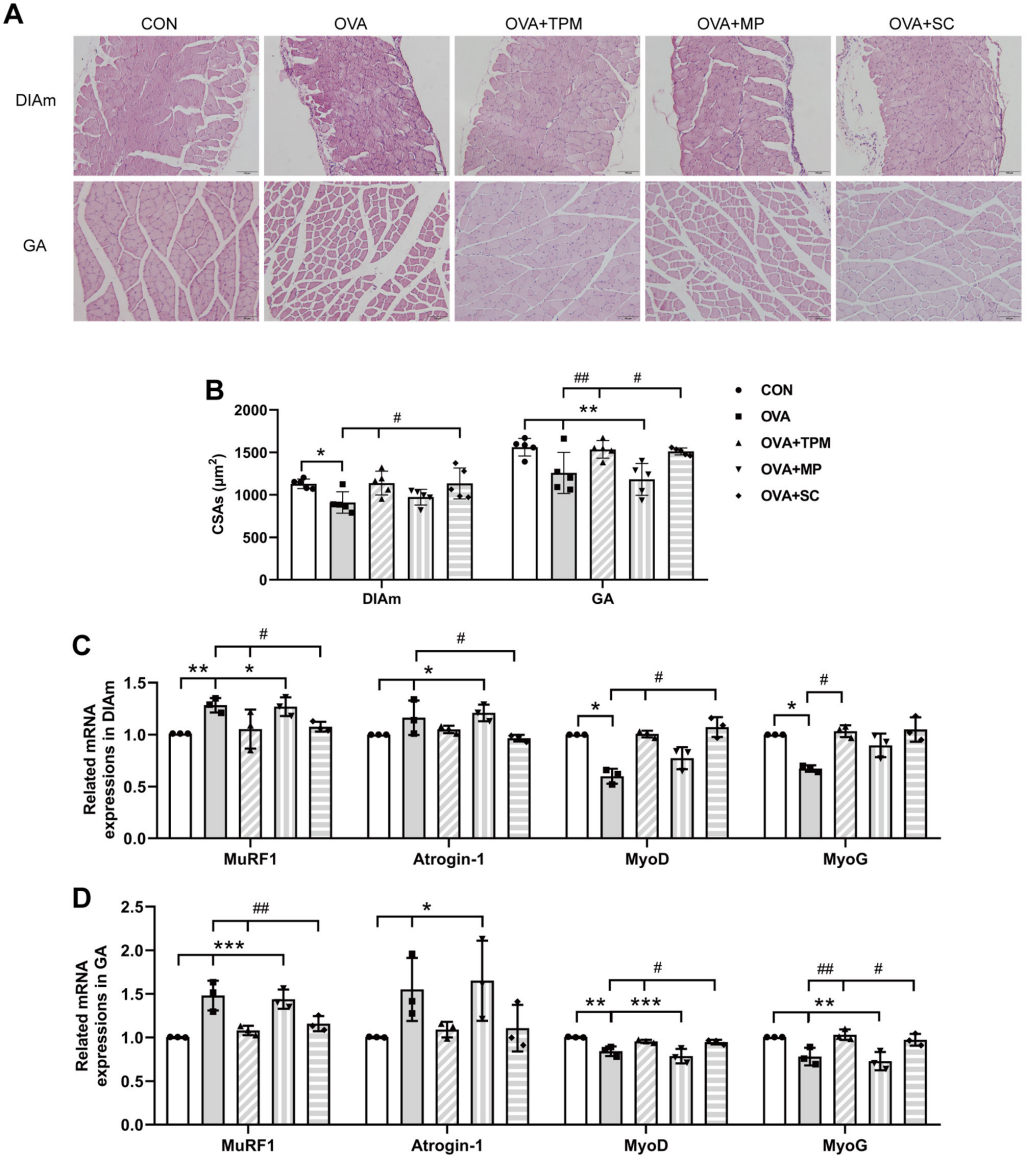
SM mass and related gene expressions in DIAm and GA. **(A, B)** Representative pictures of HE staining and CSAs of DIAm and GA (Magnification × 200, scale = 100 μm). **(C, D)** MuRF1, Atrogin-1, MyoD and MyoG mRNA expressions in DIAm and GA assessed by qRT-PCR. All data are expressed as mean ± SD (*n* = 5 for HE; *n* = 3 for qRT-PCR). ^*^P < 0.05, ^**^P < 0.01, ^***^P < 0.001, compared with the CON group. ^#^*P* < 0.05, ^##^*P* < 0.01, compared with the OVA group. CON, control; OVA, ovalbumin; TPM, traditional pediatric massage; MP, methylprednisolone sodium succinate; SC, short chain fatty acids; SM, skeletal muscle; DIAm, diaphragmatic muscle; GA, gastrocnemius; CSAs, cross sectional areas; MuRF1, muscle ring finger 1; MyoD, myogenic differentiation; MyoG, myogenin; HE, hematoxylin and eosin; qRT-PCR, quantitative real-time polymerase chain reaction.

Compared with the CON group, OVA exposure significantly reduced the CSAs of DIAm and GA fibers in adolescent rats (*P* < 0.05 and *P* < 0.01; Figures 4A, B). We further investigated these gene expressions that closely related with the degradation and synthesis of SM protein. For DIAm, OVA exposure significantly increased the gene expressions of MuRF1 and atrogin-1 (*P* < 0.01 and *P* < 0.05; Figure 4C), along with decreasing gene expressions of MyoD and MyoG (both *P* < 0.05; Figure 4C). For GA, OVA exposure also significantly increased the gene expressions of MuRF1 and atrogin-1 (*P* < 0.001 and *P* < 0.05; Figure 4D), along with decreasing the gene expressions of MyoD and MyoG (both *P* < 0.05; Figure 4D).

**Figure 5:**
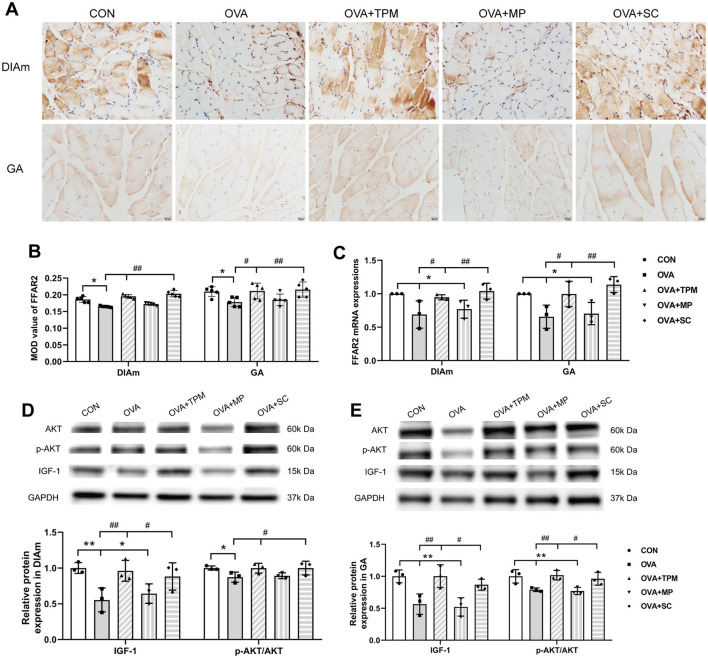
Expressions of FFAR2 and IGF-1/AKT pathway in DiaM and GA. **(A, B)** Representative pictures of IHC staining and protein expression of FFAR2 (Magnification × 400, scale = 20 μm). **(C)** FFAR2 mRNA expression assessed by qRT-PCR. **(D, E)** related protein expressions of IGF-1/AKT pathway in DiaM and GA assessed by WB. All data are expressed as mean ± SD (*n* = 5 for IHC; *n* = 3 for qRT-PCR; *n* = 3 for WB). ^*^P < 0.05, ^**^P < 0.01, compared with the CON group. ^#^*P* < 0.05, ^##^*P* < 0.01, compared with the OVA group. CON, control; OVA, ovalbumin; TPM, traditional pediatric massage; MP, methylprednisolone sodium succinate; SC, short chain fatty acids; DiaM, diaphragmatic muscle; GA, gastrocnemius; MOD, mean optical density; FFAR2, free fatty acid receptor 2; IGF-1, Insulin-like growth factor-1; AKT, protein kinase B (PKB, also called AKT); p-AKT, phosphorylated AKT; IHC, immunohistochemical; qRT-PCR, quantitative real-time polymerase chain reaction; WB, Western blotting.

A series of studies suggest that SCFAs or FFAR2 can activate IGF-1/AKT pathway to improve the loss of SM mass (Lahiri et al., 2019; Tang et al., 2022). We further investigated whether the retarded growth of SM mass was also related with FFAR2-IGF-1/AKT pathway in OVA-exposed adolescent rats. Consistent with reduced SCFAs production, OVA exposure decreased protein and gene expressions of FFAR2 in the DIAm (both *P* < 0.05; Figures 5A–C) and in GA (both *P* < 0.05; Figures 5A–C), compared with the CON group. Moreover, OVA exposure inhibited the activity of IGF-1/AKT pathway, manifested as lower protein expressions of IGF-1 and lower activities of AKT signaling in the DIAm (*P* < 0.01 and *P* < 0.05; Figure 5D) and in the GA (both *P* < 0.01; Figure 5E), compared with the CON group.

Compared with the OVA group, TPM significantly enhanced the CSAs of DIAm and GA fibers (*P* < 0.05 and *P* < 0.01; Figure 4B), corrected the abnormal gene expressions of MuRF1, atrogin-1, MyoD and MyoG in DIAm (*P* < 0.05, *P* > 0.05, *P* < 0.05 and *P* < 0.05; Figure 4C) and in GA (*P* < 0.01, *P* > 0.05, *P* < 0.05, *P* < 0.01; Figure 4D). Moreover, TPM efficiently enhanced the protein (*P* < 0.01 and *P* < 0.05; Figure 5B) and gene (both *P* < 0.05; Figure 5C) expressions of FFAR2, enhanced IGF-1 protein expressions and the activities of AKT signaling in the DIAm (*P* < 0.01 and *P* < 0.05; Figure 5D) and in the GA (both *P* < 0.01; Figure 5E).

### 3.4 SCFAs supplement increased SM mass via activating FFAR2-IGF-1/AKT pathway in the OVA-exposed adolescent rats

To investigate whether SCFAs could boost SM mass via activating FFAR2-IGF-1/AKT pathway in OVA-exposed adolescent rats, we observed the effect and potential mechanism of SCFAs, the strong agonists of FFAR2. The results showed that SCFAs significantly decreased IL-4 and IL-1β concentrations (*P* < 0.001 and *P* < 0.05; Figures 2B, C), reduced Penh and increased MV values (*P* < 0.05 and *P* < 0.01; Figures 2D, E), although it did not significantly decrease OVA-sIgE concentration (*P* > 0.05, Figure 2A) in OVA-exposed adolescent rats. SCFAs also significantly increased Shannon index and observed species of GM (*P* < 0.01 and *P* < 0.05; Figures 3A, B) and corrected the abnormal abundances of class Clostridia and class Bacilli, family Lactobacillaceae and genus Lactobacillus (*P* < 0.05, *P* < 0.01, *P* < 0.001 and *P* < 0.001; Figures 3D–F). Moreover, SCFAs enhanced the protein (both *P* < 0.01; Figure 5B) and gene (both *P* < 0.01; Figure 5C) expressions of FFAR2, increased IGF-1 protein expressions (both *P* < 0.05; Figures 5D, E), activated AKT signaling (both *P* < 0.05; Figures 5D, E) and prevented the decrease in the CSAs of DIAm and GA fibers (both *P* < 0.05; Figure 4B), along with inhibiting the abnormal gene expressions of MuRF1, atrogin-1, MyoD and MyoG in the DIAm (*P* < 0.05, *P* < 0.05, *P* < 0.05 and *P* > 0.05; Figure 4C) and in the GA (*P* < 0.01, *P* > 0.05, *P* < 0.05 and *P* < 0.05; Figure 4D).

Therefore, these results above suggest that TPM enhanced the growth of SM mass in OVA-exposed adolescent rats, which was related with regulating GM, enhancing SCFAs production and activating FFAR2-IGF-1/AKT pathway.

### 3.5 MP alleviated AAI without improving SM mass or GM dysbiosis in the OVA-exposed adolescent rats

MP significantly decreased IL-4 and IL-1β concentrations (*P* < 0.01 and *P* < 0.05; Figures 2B, C), non-significantly decreased OVA-sIgE concentration (*P* > 0.05; Figure 2A), reduced Penh value (*P* < 0.05; Figure 2D), but not increased MV value (*P* > 0.05; Figure 2E) in OVA-exposed adolescent rats. Moreover, MP did not improve GM dysbiosis. Compared with the OVA group, MP did not significantly increase Shannon index and observed species of GM (both *P* > 0.05; Figures 3A, B) or corrected the abnormal abundances of class Clostridia, class Bacilli, family Lactobacillace and genus Lactobacillus respectively (all *P* > 0.05; Figures 3D–F), which is consistent with not enhancing SCFAs concentrations (all *P* > 0.05; Figure 3G). Consistent with SCFAs results, MP did not increase the CSAs of DIAm and GA fibers (both *P* > 0.05; Figure 4B) or reverse the abnormal gene expressions of MuRF1, atrogin-1, MyoD and MyoG in the DIAm (all *P* > 0.05; Figure 4C) or in the GA (all *P* > 0.05; Figure 4D). Moreover, MP did not enhance the protein or gene expressions of FFAR2 (all *P* > 0.05; Figures 5B, C) or activate IGF-1/AKT pathway (all *P* > 0.05; Figures 5D, E) in the DIAm and GA, compared with the OVA group.

## 4 Discussion

In this study, asthma model was successfully established by OVA exposure (sensitization and challenge) in adolescent rats, where a retarded growth of SM mass and GM dysbiosis also coexisted. Meanwhile, the production of SCFAs, FFAR2 expressions and IGF-1/AKT pathway in SM were inhibited in OVA-exposed adolescent rats. TPM enhanced SM mass, improved AAI and PF, which was related with regulating GM, enhancing SCFAs production and activating FFAR2-IGF-1/AKT pathway in SM of OVA-exposed adolescent rats.

Asthma is the most common chronic disease in school-aged children (Lizano-Barrantes et al., 2024; (GINA), 2024-05-22.). Children with poorly controlled asthma always have a retarded growth of SM mass and strength (Villa et al., 2011; Latorre-Román et al., 2014; Papurcu et al., 2021; Silva et al., 2021; Zhang et al., 2022; Gokcek et al., 2023; Kankaanranta and Ilmarinen, 2023), along with the weakness and dysfunction of respiratory muscle, which can be offset by enhancing inspiratory muscle mass (de Bruin et al., 1997; Silva et al., 2013; Hellebrandová et al., 2016; Lista-Paz et al., 2023). Therefore, enhancing SM mass is beneficial to PF, which is the key goal of long-term control in children asthma (GINA, 2024). Besides the benefit to PF, enhancing SM mass in asthmatic children is also essential to their normal physical activity and metabolism regulation (Frontera and Ochala, 2015; Argilés et al., 2016). DIAm, as the most important respiratory muscle, is essential for inspiration and also generates high pressures required for expulsive efforts (Fogarty et al., 2021; Lista-Paz et al., 2023).

IGF-1 is a key up-stream molecule for activating AKT signaling, which plays an essential role in the growth and maintenance of SM mass (Stitt et al., 2004; Sandri, 2013; Frontera and Ochala, 2015). Activation of IGF-1/AKT pathway can promote myogenesis and myotube hypertrophy via triggering the expressions of myogenic transcription factors, such as MyoD and MyoG (Schiaffino et al., 2013; Frontera and Ochala, 2015). Meanwhile, IGF-1/AKT activation can also block protein degradation by inhibiting UPS (Schiaffino et al., 2013). In SM, UPS involves in proteasome-dependent degradation, which is catalyzed by the E3 enzyme, majorly including atrogin-1 and MuRF1 (Schiaffino et al., 2013; Yin et al., 2021). Childhood and adolescence are critical periods for skeletal muscle growth and development, when IGF-1 secretion is in the peak period, especially in adolescence (Loomba-Albrecht and Styne, 2009; Orsso et al., 2019; Faienza et al., 2023). However, IGF-1 expression can be inhibited in children with chronic diseases due to various factors, such as chronic inflammation, sedentary life, GCs treatment (Schiaffino et al., 2013; Chen et al., 2023). The findings of this study demonstrated that IGF-1/AKT pathway was inhibited in SM in adolescent rats of asthma model, consistent with the retarded growth of SM mass.

Lactobacilli are well known to have beneficial effects on health and immunity and are widely used as probiotics (Balamurugan et al., 2008). Lactate produced by Lactobacillus provides an unfavorable environment for the growth of many pathogenic bacteria (Wopereis et al., 2014; Ilinskaya et al., 2017). It is intriguing that abnormally higher abundance of Lactobacillus existed in adolescent rats of asthma model, which was also negatively associated with GM diversity in the present study. Our previous study also demonstrated the same result, where the adolescent rats were exposed to OVA in a shorter period (Zhu et al., 2020). Meanwhile, higher abundance of Lactobacillus was also associated with less production of SCFAs in the present study. It is well known that the diversity of GM is essential to a consistently healthy signature of SCFAs (Bauer and Thiele, 2018), which contributes to immunity balance of Th1/Th2 and anti-inflammation (Cummings et al., 1987; Bauer and Thiele, 2018; Patrick et al., 2020). Some studies have reported higher abundance of Lactobacillus in gastrointestinal disorders and suggested to use it as probiotics with caution (Wang et al., 2014; Han et al., 2019). Moreover, lower butyrate and higher lactate levels were reported to be associated with high risk for infantile allergy (Wopereis et al., 2018). A meta-analysis of clinical trials also suggested that administration of Lactobacillus acidophilus, compared with other strains, was associated with a higher risk of atopic sensitization (Elazab et al., 2013). A previous study also reported that there were ongoing changes in the composition of the fecal bacterial flora through childhood and adolescence, which was characterized by a rapid decline in Lactobacillus and possibly growing prominence of other bacteria (Balamurugan et al., 2008). Lactobacillus is typically abundant in the early colonization stage before being replaced by other anaerobic bacteria (Wopereis et al., 2018). Therefore, the present finding indicates that excessive Lactobacillus might impair the homeostasis of GM and inhibit survival of other bacterial taxa in the gut. It can also explain the reduced diversity of GM and reduced production of SCFAs.

As we know, most of SCFAs are absorbed by colonocytes and the rest enter the blood circulation through the liver, which then exert the local and systematical functions of anti-inflammation and immunity regulation (Cummings et al., 1987; Levy et al., 2017; Bauer and Thiele, 2018; Patrick et al., 2020; Gill et al., 2021). Meanwhile, More and more evidences from clinical and experimental studies support that GM dysbiosis and reduced SCFAs production can lead to the declined SM mass and strength via down-regulating IGF-1 expression and AKT signaling activation in SM, which can be corrected by SCFAs supplement or FFAR2 agonist (Lahiri et al., 2019; Lv et al., 2021; Tang et al., 2022). In the present study, consistent with GM dysbiosis and reduced SCFAs production, OVA exposure reduced the mass of DIAm and GA and inhibited the activity of FFAR2-IGF-1/AKT pathway, which were corrected by SCFAs supplement. Therefore, combined with previous studies, the present finding suggests that SCFAs may boost the growth of SM mass via relieving systematic inflammation and specifically targeting at SM via FFAR2-IGF-1/AKT pathway.

TPM can be applied on the meridians and acupoints on the abdomen, back and limbs for the regulation of visceral function and immunity based on meridian theory. A previous study demonstrated that TPM, including rubbing abdomen and pressing acupoints on the abdomen and limbs, could regulate GM to improve ulcerative colitis in mice (Wang et al., 2022). TPM is also often applied on the back, where Du vessel and bladder meridian run. In TPM clinics, back pinching manipulation is very popular in clinic and homecare for children, which is always combined with gentle manipulation like back pushing to achieve desirable effects. Back pinching manipulation produces pressing and stretching stimulation on the skin surface and the subcutaneous fascia simultaneously, which can produce moderate stimulation. Back pushing manipulation produces gentle and slow stimulation and acts majorly on the skin surface. TCM believes that stimulating Du vessel and bladder meridian can regulate visceral functions, including intestinal function, which may help GM homeostasis (Wu et al., 2024). The present study suggested that moderate stimulation combined gentle stimulation had a desirable effect on AAI alleviation and GM regulation. Moreover, this present study further demonstrates that TPM enhanced SCFAs production and activating FFAR2-IGF-1/AKT pathway, which contributed to enhancing the mass of DIAm and GA. Therefore, this present study may provide some scientific support for understanding diverse effects of TPM from the perspective of GM regulation.

MP is a commonly used glucocorticoid (GC), which is believed to induce the retarded growth or loss of SM mass via impairing the balance of protein synthesis and degradation (Schiaffino et al., 2013). In this study, we also found that MP did not significantly improve GM dysbiosis, SCFAs production and FFAR2 expression, although it significantly alleviated AAI and AHR. It indicates that chronic application of MP might also disturb GM, which may be partially related with inhibited IGF-1/AKT pathway and retarded growth of SM mass. Therefore, this study may also provide a new thought to improve the side effect of GCs on SM mass by GM regulation and enhancing SCFAs production.

## 5 Conclusion

This present study indicated that the retarded growth of SM mass and the inhibition of IGF-1/AKT pathway existed in OVA-exposed adolescent rats, which was related with GM dysbiosis, reduced SCFAs production and inhibited FFAR2 expressions in SM. TPM efficiently enhanced SM mass, at least, partially via regulating GM, enhancing SCFAs production and activating FFAR2-IGF-1/AKT pathway.

## 6 Limitations

There are several limitations in this study. Firstly, we did not apply specific FFAR2 agonist to determine the key role on activating IGF-1/AKT pathway, since SCFAs can also activate free fatty acid receptor 3 (also known as GPR41). Secondly, we did not detect IGF-1 expression in the liver or its concentration in blood circulation, which may arrive in SM and play a role in the growth of SM. IGF-1 in SM can be also originated from blood circulation, which is majorly secreted by the liver, although local IGF-1 secretion proves to play a key role in tissue development (Martín et al., 2021). Thirdly, we didn't investigate the efficiency and potential mechanism of the combined treatment of MP and TPM, which is a common choice for children with asthma. Therefore, the future study warrants further investigation.

## 7 Clinical significance

It is well known that TPM can be provided either by professionals in clinics, or by parents at home (Wu et al., 2024). Therefore, as a feasible, effective and safe complementary method, TPM can be applied on asthmatic children by parents under the guide of professional staffs for a better control besides routine treatment.

## Data Availability

The datasets presented in this study can be found in online repositories. The names of the repository/repositories and accession number(s) can be found below: https://www.ncbi.nlm.nih.gov/, PRJNA1198868.
